# Three-Year Outcomes in Kidney Transplant Recipients Switched From Calcineurin Inhibitor-Based Regimens to Belatacept as a Rescue Therapy

**DOI:** 10.3389/ti.2022.10228

**Published:** 2022-04-13

**Authors:** Antoine Morel, Léa Hoisnard, Caroline Dudreuilh, Anissa Moktefi, David Kheav, Ana Pimentel, Hamza Sakhi, David Mokrani, Philippe Attias, Karim El Sakhawi, Cécile Maud Champy, Philippe Remy, Emilie Sbidian, Philippe Grimbert, Marie Matignon

**Affiliations:** ^1^ Nephrology and Renal Transplantation Department, AP-HP (Assistance Publique-Hôpitaux de Paris), Hôpitaux Universitaires Henri Mondor, Créteil, France; ^2^ AP-HP (Assistance Publique-Hôpitaux de Paris), Hôpitaux Universitaires Henri Mondor, Centre d’Investigation Clinique and Fédération Hospitalo-Universitaire TRUE (InnovaTive theRapy for immUne disordErs), Créteil, France; ^3^ Université Paris Est Créteil (UPEC), EpiDermE (Epidemiology in Dermatology and Evaluation of therapeutics), Créteil, France; ^4^ Groupe Hospitalier Henri-Mondor/Albert-Chenevier, Pathology Department, AP-HP (Assistance Publique-Hôpitaux de Paris), Créteil, France; ^5^ Institut National de la Santé et de la Recherche Médicale (INSERM) U955, Institut Mondor de Recherche Biomédicale (IMRB), Université Paris-Est Créteil, Créteil, France; ^6^ AP-HP (Assistance Publique-Hôpitaux de Paris), Laboratoire Régional d’histocompatibilité, Hôpital Saint Louis, Vellefaux, Paris; ^7^ Groupe Hospitalier Henri-Mondor/Albert Chenevier, Urology department, AP-HP (Assistance Publique-Hôpitaux de Paris), Hôpitaux Universitaires Henri Mondor, Créteil, France; ^8^ Department of Dermatology, AP-HP (Assistance Publique-Hôpitaux de Paris), Hôpitaux Universitaires Henri Mondor, Créteil, France; ^9^ INSERM, Centre d’Investigation Clinique 1430, Créteil, France; ^10^ AP-HP (Assistance Publique-Hôpitaux de Paris), Hôpitaux Universitaires Henri Mondor, CIC biotherapy, Créteil, France

**Keywords:** kidney transplantation, transplant outcomes, belatacept conversion, rescue therapy, CNI

## Abstract

**Background:** The long-term benefits of conversion from calcineurin inhibitors (CNIs) to belatacept in kidney transplant recipients (KTr) are poorly documented*.*

**Methods:** A single-center retrospective work to study first-time CNI to belatacept conversion as a rescue therapy [eGFR <30 ml/min/1.73 m^2^, chronic histological lesions, or CNI-induced thrombotic microangiopathy (TMA)]. Patient and kidney allograft survivals, eGFR, severe adverse events, donor-specific antibodies (DSA), and histological data were recorded over 36 months after conversion.

**Results:** We included N = 115 KTr. The leading cause for switching was chronic histological lesions with non-optimal eGFR (56.5%). Three years after conversion, patient, and death-censored kidney allograft survivals were 88% and 92%, respectively, eGFR increased significantly from 31.5 ± 17.5 to 36.7 ± 15.7 ml/min/1.73 m^2^ (*p* < 0.01), the rejection rate was 10.4%, OI incidence was 5.2 (2.9–7.6) per 100 person-years. Older age was associated with death, eGFR was not associated with death nor allograft loss. No patient developed *dn*DSA at M36 after conversion. CNI-induced TMA disappeared in all cases without eculizumab use. Microvascular inflammation and chronic lesions remained stable.

**Conclusion:** Post-KT conversion from CNIs to belatacept, as rescue therapy, is safe and beneficial irrespective of the switch timing and could represent a good compromise facing organ shortage. Age and eGFR at conversion should be considered in the decision whether to switch.

## Graphical Abstract

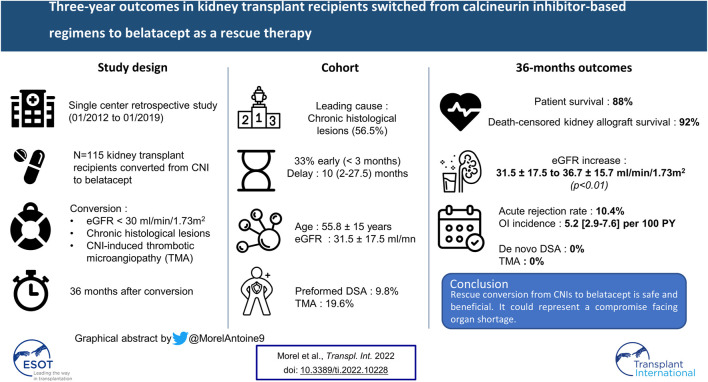

## Introduction

Despite the improvement of kidney allograft short-term survival with conventional immunosuppressive agents, allograft long-term survival has not increased as expected (1). One of the main reasons is the growing proportion of expanded criteria donors (ECD) in kidney transplantation (KT) (2). Calcineurin inhibitors (CNIs) are the standard long-term immunosuppression therapy in kidney transplant recipients (KTr), albeit they could contribute to acute and chronic impairment of kidney allograft function, especially in patients with chronic histological damage (2–5,6,8). Therefore, new immunosuppressive strategies are needed to preserve kidney allograft function and improve the graft long-term survival (6).

Belatacept is a CD80/CD86—CD28 T-cell selective costimulation blocker developed to counteract CNI-induced nephrotoxicity. Two prospective randomized trials (BENEFIT and BENEFIT-EXT study) reported long-term safety and efficacy of *de novo* belatacept treatment coupled with the improvement of estimated glomerular filtration rate (eGFR), and similar patient or kidney allograft survival in comparison with cyclosporine (8–11).

Since, growing evidence has suggested shifting KTr from CNIs-based regimen to belatacept, especially in those with low graft function or chronic histological lesions where belatacept is used as a rescue therapy (12–15). Additionally, it might be effective in sensitized kidney allograft recipients with preformed DSA (13–16). There is very little knowledge on the outcome of patients switched to belatacept after 1 year of follow-up, and what data there is often come from small sample cohorts, without DSA nor histological evolution analysis after the switch (8, 16).

In this study, we assessed the safety and tolerability of belatacept treatment as a rescue therapy up to 3 years after switching from CNIs. Additionally, we analyzed kidney allograft function, patient and kidney allograft survival, and major outcomes after switching to belatacept and its effects on both DSA and kidney allograft histology changes.

## Methods

### Patients and Study Design

In this retrospective monocentric study, we included all adults KTr converted for the first time from CNI-based immunosuppressive regimen to belatacept from January 2012 to January 2019. Patients who tested negative for EBV before transplantation, pregnant women, or women not on any contraceptive methods were not included since they were not eligible to receive belatacept treatment.

The KTr Cohort Was Approved by IRB #00003835.

### Interventions

Early and late conversion groups were defined according to the time of conversion from KT to first belatacept infusion: < 3 months or >3 months, respectively. In early-stage conversion, CNIs were stopped at day 1, and KTr were given 10 mg/kg belatacept infusions at days 1, 5, 14, and 28, and weeks 8 and 12, and then 5 mg/kg from week 16 onwards, every 4 weeks. In late-stage conversion, CNIs were stepped down to 50% at day 14 and stopped at day 28 after conversion, and belatacept infusions were given at 5 mg/kg at day 1, 14, 28, and then every 4 weeks thereafter (8).

### Study Endpoints

The primary endpoint was the safety and tolerability of belatacept treatment. Major adverse events were defined as patient death and kidney allograft loss. Follow-up continued till August 30, 2021 or the date when a major adverse event occurred. Other severe adverse events (SAE) included community-acquired infections requiring hospitalization, OIs, acute rejections, and neoplasia.

Secondary endpoints were: 1) eGFR and urine protein/creatinine ratio (UPCR) evolution up to 3 years after conversion, 2) identification of different clusters of eGFR trajectory after conversion, 3) metabolic parameters (LDL, HDL-cholesterol, triglycerides concentration, HbA1C) and blood pressure profile evolution, 4) CMV or BK viremia, 5) pre-existing and *dn*DSA evolution, and 6) histological lesions evolution.

### Community-Acquired Infection, Opportunistic Infection Definitions, and Anti-microbial Prophylaxis

Community-acquired infections were considered only in case of hospitalization. Opportunistic Infection (OIs) were defined according to the current literature and international guidelines (18). OIs caused by the following pathogens were considered:

- Bacteria: *Mycobacterium* sp., *Listeria* monocytogenes, and *Nocardia* sp.
- Viruses: Cytomegalovirus (CMV), Varicella-Zoster virus (VZV), Human Herpes Virus-8 (HHV8), Norovirus, BK virus nephropathy, and JC virus.
- Fungi: Candida spp., Cryptococcus spp., invasive molds, and Pneumocystis jirovecii.
- Parasites: Toxoplasma gondii, Microsporidium sp., Cryptosporidium sp., Leishmania sp.

Patients were screened for BK viremia once a month during the first 3 months after KT, then every 3 months till the end of the first year, and every year till the end of year 5. After switching to belatacept, BKV was monitored every 3 months during the first year then once a year up to 5 years after KT. CMV prophylaxis followed the international guidelines: valganciclovir for 6 months in high-risk patients CMV D^+^/R^−^ and 3 months in intermediate-risk patients CMV D^+^/R^+^ or CMV D^−^/R^+^ (19). Pneumocystis prophylaxis (Trimethoprim + Sulfamethoxazole) was administered during the first post-KT year.

### Variables

Demographic characteristics, medical data, and laboratory samples, in particular eGFR, UPCR, and DSA, were collected at the time of transplantation, at the time of conversion, and during belatacept treatment (3, 12, 24, 36, 48, and 60 months).

GFR was estimated using the Modification of diet in renal disease (MDRD) formula (20). Indications for switching to belatacept were recorded. Chronic histological lesions associated with suboptimal allograft function was defined as eGFR <30 ml/min/1.73 m^2^ and histological lesions associating ci + ct ≥ 3 and/or cv + ah ≥ 2.

Delayed graft function (DGF) was defined as the need for dialysis within 7 days after transplantation (21). Allograft loss was defined as the need for long-term dialysis and/or retransplantation.

### Anti-HLA Antibody Screening

High-resolution DNA typing was performed in donors and recipients (HLA-A, HLA-B, Cw, HLA-DR, HLA-DQ, or HLA-DP) at the time of KT. All serum samples were assessed for the presence of circulating preformed DSA and *de novo* DSA (*dn*DSA) on all HLA loci (HLA-A, HLA-B, Cw, HLA-DR, HLA-DQ, or HLA-DP) at the time of KT, at conversion, at 3, 12, 24, 36, 48, and 60 months using high-resolution Luminex SAB assay technology (One Lambda, Inc., Canoga Park, CA, United States) on Luminex platform. All beads with MFI >1,000 were considered positive (22). *Dn*DSA were considered positive if MFI was higher than 1,000 at two time points.

Naturally existing DSA antibodies (i.e., presence of DSA in patients with no past immunizing events such as transfusion or pregnancy or having a previous transplant at the time of KT), as well as IgM DSA, were not considered in our study (23).

### Histological Analysis

Patients underwent for-cause or protocol kidney allograft biopsies. Acute and chronic histological lesions were described according to the updated Banff classification (24).

### Statistical Analysis

Continuous variables were presented in mean (standard deviation, SD) or median (Interquartile range, IQR) as appropriate, and categorical variables in number and percentage. We used *t*-test or Wilcoxon test for continuous variables, and Chi-2 or Fisher exact tests for categorical variables. Paired *t*-test was used to compare quantitative variables at two different time points. In patients who had at least two kidney biopsies (before and after conversion), paired comparisons of histological lesions were performed using Mc Nemar test or binomial test.

Time to death and to allograft loss, and survival without rejection after conversion (censored for death, kidney allograft loss, and belatacept withdrawal) were displayed with Kaplan Meier curves. Hazard ratios were estimated by the Cox regression model. Incident rates of SAEs were estimated per 100 person-years (PY) with their confidence interval and the inter-group ratio of such incidence rate.

Sensitivity analyses of eGFR and proteinuria evolution after conversion to belatacept were performed with imputations of missing data regarding allograft loss (as 6 ml/min/1.73 m^2^) alone then death and allograft loss together. However, data missed because of belatacept treatment interruption over the 3-year period were not imputed since the causes of interruption were multiple.

To identify clusters of eGFR trajectories, we used the *k*-means method relying on expectation-maximization algorithms. Sensitivity analyses were performed for mean eGFR at different time points and eGFR trajectories. Missing data due to graft loss and/or death were imputed as 6 ml/min/1.73 m^2^.

A *p*-value <0.05 was considered significant. Tests were two-tailed. Statistical analyses were carried out using R 3.6.2.

## Results

From January 2012 to 01/2019, 115 patients underwent first-time switch from CNI to belatacept, of whom 38 (33%) had an early-stage switch, and 76 patients (66.1%) were men.

At the time of transplantation (Table 1), the mean age was 55.8 ± 15 years old. Almost all donors were deceased [N = 108 (93.9%)], mainly ECD [N = 69 (60%)], and 61.5 ± 15 years old. All recipients who received induction immunosuppressive therapy, such as anti-interleukin-2 receptor [N = 61/115 (53%)], and N = 11/115 (9.8%) had pretransplant DSA. Maintenance immunosuppressive therapy included CNIs (100%), mycophenolic acid (MPA) (82.6%), and steroids (100%). Of more, N = 22/115 (19.1%) patients were at high risk for CMV transmission (D^+^/R^−^).

**TABLE 1:** Clinical and biological characteristics at the time of transplantation.

Variables	Whole Cohort, N = 115	Late Switch, N = 77	Early Switch, N = 38
Recipient characteristics			
Age, mean ± SD	55.8 (15.0)	53.9 (15.0)	60.0 (14.3)
Gender (Male), N (%)	76 (66.1)	51 (66.2)	25 (65.8)
Hemodialysis, N (%)	106 (92.2)	74 (96.1)	32 (84.2)
Previous KT, N (%)	15 (13.0)	13 (16.9)	2 (5.3)
Initial nephropathy			
Glomerulopathy, N (%)	24 (20.9)	15 (19.5)	9 (23.7)
Diabetes mellitus, N (%)	18 (15.7)	9 (11.7)	9 (23.7)
Nephroangiosclerosis, N (%)	11 (9.6)	8 (10.4)	3 (7.9)
Genetic, N (%)	10 (8.7)	8 (10.4)	2 (5.3)
Autoimmune disease, N (%)	4 (3.5)	3 (3.9)	1 (2.6)
Other, N (%)	22 (19.1)	15 (19.5)	7 (18.4)
Undetermined, N (%)	26 (22.6)	19 (24.7)	7 (18.4)
Donor			
Age, mean ± SD	61.5 (15)	60.6 (14.29)	63.4 (16.39)
Living donor, N (%)	7 (6.1)	5 (6.5)	2 (5.3)
Extended criteria donor, N (%)	69 (60)	46 (59.7)	23 (60.5)
Donor/recipient CMV status			
D+/R+, N (%)	51 (44.3)	36 (48.8)	15 (39.5)
D+/R-, N (%)	22 (19.1)	16 (20.8)	6 (15.8)
D-/R+, N (%)	34 (29.6)	21 (27.3)	13 (34.2)
D-/R-, N (%)	8 (7)	4 (5.2)	4 (10.5)
Kidney transplant characteristics			
Anti HLA donor specific antibodies, N (%)	11 (9.8)	6 (7.9)	5 (13.9)
Cold ischemia time, hours N = 112, N (%)	18.1 (5.7)	18.2 (5.5)	17.7 (6)
Delayed graft function, N (%)	51 (44.3)	31 (40.3)	20 (52.6)
Induction immunosuppressive therapy			
Anti-interleukin 2 receptor, N (%)	61 (53)	42 (54.5)	19 (50)
Antithymocyte globulin, N (%)	54 (47)	35 (45.5)	19 (50)
Maintenance immunosuppressive therapy			
Calcineurin inhibitors, N (%)			
Cyclosporine	20 (17.4)	16 (20.8)	4 (10.5)
Tacrolimus	95 (82.6)	61 (79.2)	34 (89.5)
Mycophenolic acid (MPA), N (%)	95 (82.6)	66 (85.7)	29 (76.3)
mTOR inhibitors, N (%)	19 (16.5)	11 (14.3)	8 (21.1)
Steroids	115 (100)	77 (100)	38 (100)

KT, Kidney transplantation; mTOR, Mammalian target of rapamycin.

At the time of conversion (Table 2), 10 (2–27.5) months after KT, class I and II DSA were detected in 8/115 (7%) and 12/115 (10.4%) patients, respectively. In the late-switch group, the main cause of conversion was chronic vascular histological lesions associated with non-optimal kidney allograft function (71.4%), whereas it was prolonged DGF (55.3%) in the early-switch group. Concomitant immunosuppression is provided in Table 2. A median number of anti-hypertensive drugs was 2 (1–2), levels of HbA1c, LDL, and HDL-cholesterol were 5.9 ± 0.5%, 2.1 ± 0.5 g/L, and 1 ± 0.3 g/L, respectively, (Supplementary Table S3)**)**.

**TABLE 2:** Clinical and biological characteristics at the time of conversion.

Variables	Whole Cohort, N = 115	Late Switch, N = 77	Early Switch, N = 38
Conversion time from KT, months, median (IQR)	10 (2–27.5)	17 (10–67)	1 (1–2)
Age, mean ± SD	58.6 (14.4)	57.5 (14.5)	60.8 (14.2)
Reasons for switching			
Prolonged delayed graft function, N (%)	23 (20)	2 (2.6)	21 (55.3)
Chronic histological lesions associated with suboptimal allograft function (ci + ct ≥ 3 and/or cv + ah ≥ 2), N (%)	65 (56.5)	55 (71.4)	10 (26.3)
Thrombotic microangiopathy, N (%)	21 (18.3)	17 (22.1)	4 (10.5)
Other renal causes, N (%)	4 (3.5)	1 (1.3)	3 (7.9)
Undetermined issues, N (%)	2 (1.7)	2 (2.6)	0 (0)
Kidney allograft function			
eGFR (mL/min/1.73 m^2^), mean ± SD	31.7 (17.8)	33.9 (16.9)	27.3 (19)
Urine protein/creatinine ratio >100 mg/mmol, N (%)	29 (25.9)	15 (19.7)	14 (38.9)
Drugs			
Antihypertensive drugs, median (IQR)	2 (1–2)	2 (1–2)	2 (1–2)
MPA, N (%)	104 (90.4)	69 (89.6)	35 (92.1)
500 mg per day, N (%)	12 (11.5)	11 (15.9)	1 (2.9)
1,000 mg per day, N (%)	40 (38.5)	38 (55.1)	2 (5.7)
2,000 mg per day, N (%)	40 (38.5)	9 (13.0)	31 (88.6)
Other dose, N (%)	12 (11.5)	11 (15.9)	1 (2.9)
mTOR inhibitors, N (%)	10 (8.7)	7 (9.1)	3 (7.9)
T0 level (ng/ml), median (IQR)	5.2 (4.3–6.1)	4.5 (4.2–5.3)	6.3 (5.8–6.9)
Corticosteroids, N (%)	115 (100)	77 (100)	38 (100)
5 mg per day, N (%)	84 (73.0)	75 (97.4)	9 (23.7)
10 mg per day, N (%)	31 (27.0)	2 (2.6)	29 (76.3)
Anti HLA donor specific antibodies			
Class I, N (%)	8 (7)	6 (7.8)	2 (5.3)
Class II, N (%)	12 (10.4)	12 (15.6)	0 (0)
Both class I and class II, N (%)	2 (1.7)	2 (2.6)	0 (0)
None, N (%)	97 (84.3)	61 (79.2)	36 (94.7)
Kidney biopsy (Banff lesions score)	N = 102	N = 77	N = 25
Biopsy to conversion time, days, median (IQR)	35 (92–12)	48 (118–26)	8.5 (19.8–6.2)
Acute tissue injury			
Banff lesions score ≥1 in at least one compartment, N (%)	50 (48.5)	37 (48.1)	13 (50)
Acute tubular necrosis, N (%)	20 (19.2)	11 (14.3)	9 (33.3)
Glomerulitis (g), N (%)	7 (6.7)	7 (9.1)	0 (0)
Interstitial inflammation (i), N (%)	3 (2.9)	3 (3.9)	0 (0)
Tubulitis (t), N (%)	10 (9.6)	9 (11.7)	1 (3.7)
Peri-tubular capillaritis (cpt), N (%)	3 (2.9)	2 (2.6)	1 (3.7)
Vascular inflammation (v), N (%)	0 (0)	0 (0)	0 (0)
Thrombotic microangiopathy, N (%)	21 (19.6)	17 (22.1)	4 (13.3)
g + cpt (≥2), N (%)	9 (8.7)	8 (10.4)	1 (3.7)
Chronic tissue injury			
Banff lesions score ≥1 in at least one compartment, N (%)	97 (97)	74 (98.7)	23 (92)
Transplant glomerulopathy (cg), N (%)	8 (7.8)	8 (10.4)	0 (0)
Interstitial fibrosis (ci), N (%)	89 (87.3)	68 (89.5)	21 (80.8)
Total inflammation (ti), N (%)			
Tubular atrophy (ct), N (%)	84 (82.4)	66 (86.8)	18 (69.2)
Chronic vasculopathy (cv), N (%)	67 (65.7)	48 (63.2)	19 (73.1)
Arteriolar hyalinization (ah), N (%)	80 (79.2)	61 (81.3)	19 (73.1)
IFTA (ci + ct), N (%)	93 (92.1)	71 (93.4)	22 (88.0)
ci + ct + cg + cv, median (IQR)	4 (2–5)	4 (3–6)	4 (2–4)

KT, Kidney transplantation; eGFR, Estimated glomerular filtration rate; MPA, Mycophenolic acid; mTOR, Mammalian target of rapamycin; IFTA, Interstitial fibrosis and tubular atrophy.

### Analysis at Month 36

The last follow-up checking was on August 30, 2021. Recipients were followed over 40.2 ± 30.1 months after conversion and *N* = 58/115 (51%) completed 36 months of follow-up. Of the remaining 57 patients who did not reach the third year time point, N = 26/57 discontinued belatacept (alive with functional kidney allograft), N = 13/57 died, N = 9/57 lost their KT, N = 8/57 did not complete 36 months, and N = 1/57 was lost to follow-up. Three of the study patients (N = 115; 2.6%) aged more than 70 years old were treated for less than 3 months. The first developed BK virus nephropathy a month after conversion (blood BK virus replication >6 log at the time of switch), hence the interruption of belatacept. The other two patients were switched to belatacept for arterial thrombosis and primary non-function. Both developed rapid kidney allograft failure requiring renal replacement therapy and interruption of belatacept.

**FIGURE 1:**
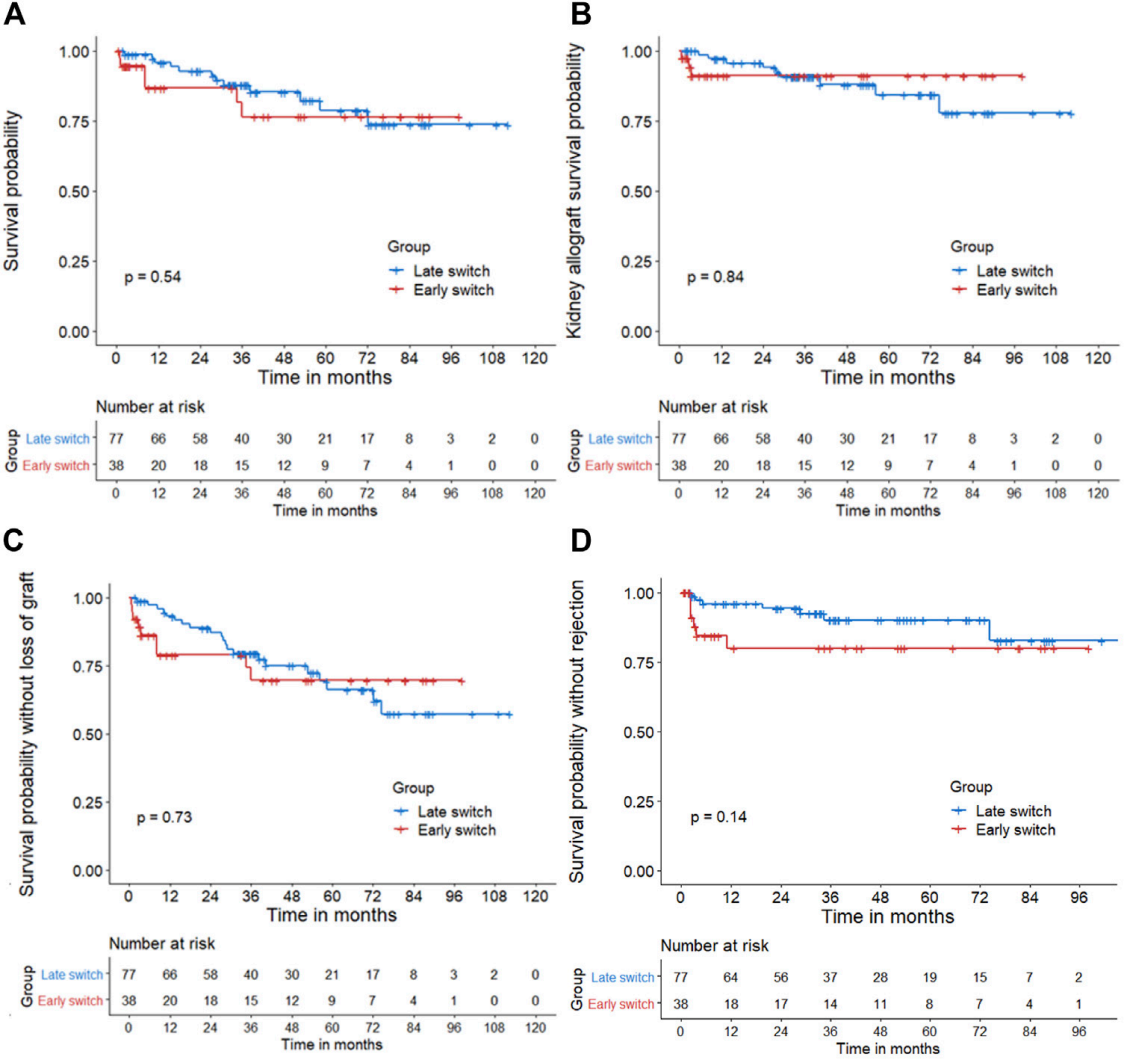
**(A)**: Patient survival—**(B)**: Death-censored kidney allograft survival—**(C)**: Global survival (using a composite outcome of the patient and death-censored kidney allograft survivals)—**(D)**: Survival without acute rejection (censored for death, kidney allograft loss, and belatacept withdrawal). Kaplan-Meier method was used to assess patient survival from time of belatacept conversion (time 0). *p*-values were measured from the log-rank test. *X*-axis: Post-conversion months. The blue curve represents the late switch group, whereas the red curve represents the early switch group. There was no statistical difference of patient, kidney allograft, global survival or survival probability without rejection between early and late conversion groups using Cox analysis (*p* = 0.54, *p* = 0.84, *p* = 0.73, and *p* = 0.14, respectively).

Three years after conversion, patient’s and death-censored kidney allograft survival rates were respectively 88% and 92%, which dropped down to 81% and 89% at year 5 (Figures 1A,B). Overall graft survival was similar between groups (Figure 1C). Age was the only significant risk factor for death after conversion in the univariate analysis [HR: 1.05 (1.01–1.1)]. None of the other factors (conversion time from KT, gender, or eGFR) was significantly associated with death or allograft loss (Supplementary Table S1). Estimated GFR significantly increased during the 36 months after conversion, from 31.5 ± 17.5 to 36.7 ± 15.7 ml/min/1.73 m^2^ (*p* < 0.01). This significant increase was confirmed in the sensitivity analysis (*p* = 0.05; Supplementary Table S2). UPCR remained stable after conversion without and with sensitivity analysis (Supplementary Table S2). HbA1c, HDL, and LDL-c serum levels significantly decreased over the 36 months after conversion (*p* < 0.01 in all parameters; Supplementary Table S3). The number of anti-hypertensive drugs and the triglycerides level remained stable (*p* = 0.87 and *p* = 0.39, respectively) (Supplementary Table S3).

### Major Adverse Events at the End of Follow-Up

At the end of follow-up, 18/115 (16%) patients died, 12/115 (14%) had allograft failure, and 31/115 (26.9%) discontinued their treatment. The main causes of death were infection (N = 11/115, 9.5%), including three cases of COVID-19, followed by cardiovascular diseases (N = 6/115, 5.2%), and neoplasia (N = 1/115, 0.9%). Allograft loss was mainly due to chronic allograft dysfunction (N = 7/115, 6.1%); other causes implied primary non-function (N = 3/115, 2.6%), chronic antibody-mediated rejection (ABMR) (N = 1/115, 0.9%), and BK virus nephritis (N = 1/115, 0.9%).

The leading cause of belatacept discontinuation was OIs episodes (*n* = 10/31, 32.3%), albeit no patient discontinued because of allograft loss or death. None of the 31 patients who had their treatment interrupted died and N = 6/31 (19.3%) lost their kidney allograft within 1 year after belatacept interruption. Reasons for kidney allograft loss in those were as follows: N = 4/6 chronic dysfunction, N = 1/6 acute ABMR, and N = 1/6 severe focal and segmental glomerulosclerosis (FSGS).

### SAE at the End of Follow-Up

At the end of the follow-up, the incidence of acute rejection was 10.4% (N = 12/115). Two patients developed another rejection episode. The most common rejection mechanism was acute T-cell mediated (TCMR) (N = 5/115, 4.3%), occurring within the first 3 months after conversion. Incidence was similar in early and late switch groups (20.0% and 9.8% respectively; *p* = 0.14) (Figure 1D). Evolution after rejection was as follows: 1) no patient died, 2) all discontinued belatacept infusion except one case with borderline lesions, and 3) one kidney allograft loss within 1 year after conversion (refractory acute ABMR).

Incidences rates of OI and community-acquired infections were 5.2 (2.9–7.6) and 15.6 (11.1–20) per 100 PY, respectively. The 19 OIs happened 10 (2–17) months after conversion and were mainly CMV disease (N = 7/115, 6.1%) and pneumocystis pneumonia (N = 5/115, 4.3%) (Table 3). BK viremia was reported in N = 11/115 (10.8%) patients and CMV reactivation in N = 27/115 (26.5%), especially in early conversion group (38.9% vs. 17.1% in late conversion group, *p* = 0.012). Among the N = 19 OI patients, the infection caused the death of N = 4/19 (21%), but no allograft loss was reported.

**TABLE 3:** Serious adverse events after conversion, incidence rates per 100 person-years (PY) of treatment exposure.

Events	Whole Cohort N = 115 N (%)	Whole Cohort N = 115 incidence per 100 PY [95% CI]	Late Switch N = 77	Early Switch N = 38	Incidence Rate Ratio [95% CI]
Rejections	12 (10.4)	3.5 [1.6–5.5]	2.6 [1.0–5.3]	6.1 [2.2–13.3]	2.36 [0.66–8.21]
Borderline	1 (0.9)				
Mixed	2 (1.8)				
Acute TCMR	5 (4.3)				
Acute ABMR	1 (0.9)				
Chronic ABMR	3 (2.6)				
Infections					
Community acquired infections	47 (40.9)	15.6 [11.1–20.0]	12.3 [8.2–17.8]	25.7 [15.5–40.1]	2.08 [1.1–2.62]
Opportunistic infections	19 (16.5)	5.2 [2.9–7.6]	5.4 [3.1–8.8]	4.8 [1.8–10.5]	0.89 [0.25–2.62]
CMV disease	7 (6.1)				
Pneumocystosis	5 (4.3)				
VZV	4 (3.5)				
Other OI	3 (2.6)				
Neoplasia	14 (12.2)	3.9 [1.9–6.0]	3.8 [1.8–7.0]	4.3 [1.2–10.9]	1.12 [0.26–3.88]
Solid malignancy	8 (7.0)				
Non-melanoma skin cancer	5 (4.3)				
Post-transplant lymphoproliferative disorder	1 (1.0)				

TCMR, T cell-mediated rejection; ABMR, Antibody-mediated rejection; CMV, Cytomegalovirus; VZV, Varicella-Zoster-Virus; OI, Opportunistic infection; PY, Person-year; CI, Confidence interval.

Malignancies were reported in 14/115 (12.2%) recipients, the incidence rate was 3.9 (1.9–6.0) per 100 PY. Most of them had solid malignancy (N = 8/115, 7%) and non-melanoma skin cancers (N = 5/115, 4.3%). We documented one case of post-transplant lymphoproliferative disorder. Of the 14 cancer patients, none stopped belatacept treatment, one died of esophagus neoplasia within 6 months of diagnosis, and two lost their kidney allograft after chronic progressive kidney allograft dysfunction.

The incidence rate ratio between late and early switch groups was similar for all of the studied SAE (e.g., acute rejection, infections, and malignancies) (Table 3).

### eGFR Trajectories After Conversion

**FIGURE 2:**
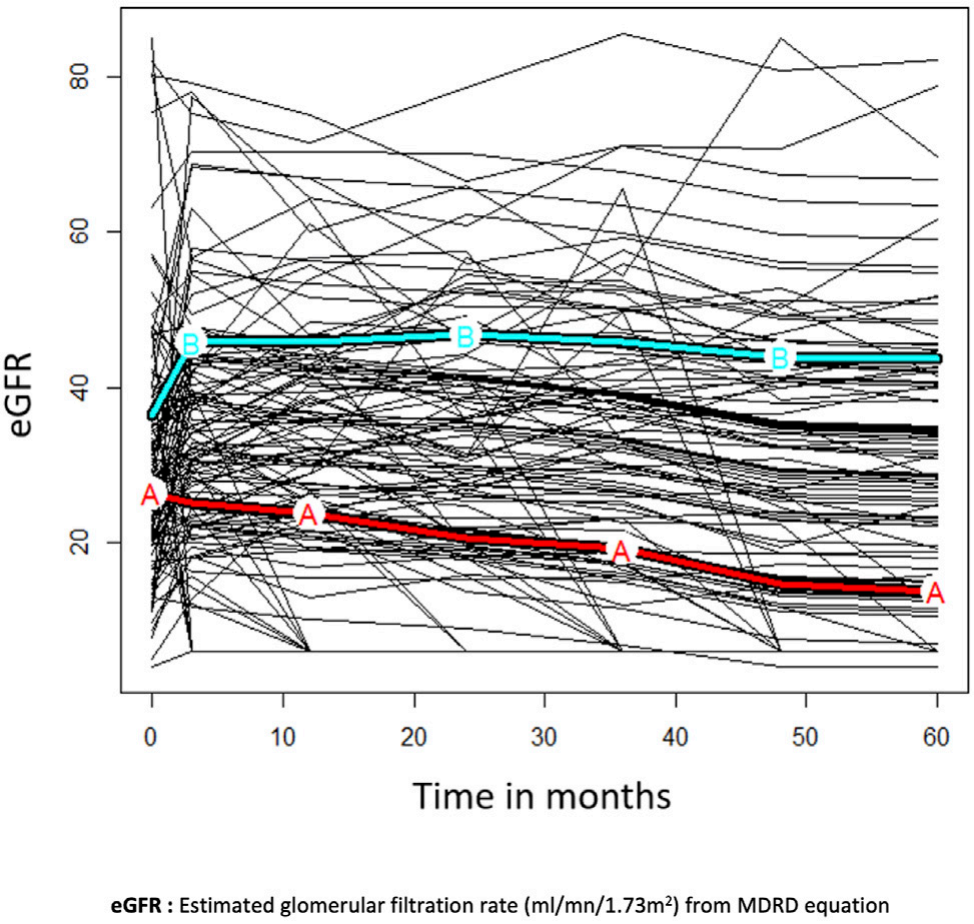
Clusters of estimated glomerular filtration rate trajectories **(A,B)** in a subgroup of N = 114 patients with at least 1 year of follow-up. Missing data were imputed at 6 ml/min for patients who died or lost their allograft. Trajectory A is represented by the red curve and trajectory B by the cyan curve. eGFR rapidly improved at three months and remained stable overtime in trajectory **(B)**. In trajectory **(A)**, eGFR progressively decreased after conversion.

Two distinct eGFR trajectories were identified in the N = 114 recipients after conversion (Figure 2): trajectory A in N = 64/114 (56.1%) KTr and trajectory B in N = 50/114 (43.9%). eGFR rapidly improved at 3 months and remained stable over time in trajectory B. In trajectory A, eGFR progressively decreased after conversion. Cluster A recipients were more likely to have renal replacement therapy before KT (*p* < 0.01), previous KT (*p* = 0.01), eGFR <30 ml/min/1.73 m^2^ at the time of conversion (<0.01) (Supplementary Table S4). Other characteristics (i.e., early or late switch, recipients’ age, and histological characteristics before conversion) did not differ significantly between trajectory clusters.

### Pre-Existing and *de novo* DSA Analysis

Before the switch, DSA was detected in N = 18/115 (15.7%) patients whose number remained stable over time after conversion (Table 4), though one developed chronic ABMR [N = 1/18 (5.6%)]. No patient developed *dn*DSA at the end of the follow-up.

**TABLE 4:** Preformed and *de novo* DSA evolution after conversion.

	Switch N (%) or Median (Q1-Q3)	M3	M12	M24	M36	M48	M60
Available data DSA	115	107	86	77	56	40	34
Pre-existing DSA	18 (15.7)	17/107 (17.2)	18/86 (20.9)	14/77 (18.2)	10/56 (17.9)	8/40 (20)	8/34 (23.5)
Class I	8 (7)	7/107 (6.5)	4/86 (4.6)	5/77 (6.5)	2/56 (3.6)	2/40 (5)	3/34 (8.8)
Class I MFI max	2,188 (1,601–2,844)		1903 (1,288–2,569)		3,302 (2,876–3,728)		
Class I MFI sum	2,388 (1807–3,841)		2,453 (2090–2,569)		3,302 (2,876–3,728)		
Class II	12 (10.4)	12/107 (11.2)	16/86 (18.6)	12/77 (15.6)	9/56 (16.2)	7/40 (1.8)	7/34 (20.6)
Class II MFI max	1769 (1,433–2,951)		1,472 (1,201–3,314)		3,273 (1957–5,092)		
Class II MFI sum	2,920 (1,642–3,178)		2027 (1,292–4,457)		3,674 (1857–5,277)		
*dn*DSA appearance	0	0 (0)	0 (0)	0 (0)	0 (0)	0 (0)	0 (0)

dnDSA, De novo Donor Specific Antibodies; MFI, Mean fluorescence intensity.

### Histological Analysis

Among the 48 patients who underwent paired kidney allograft biopsies (Table 5), the second biopsy was performed 378 (182–802) days after conversion and 60.1% were for-cause biopsies. Regarding acute tissue injuries, all CNI-associated acute thrombotic microangiopathy (TMA) lesions disappeared after conversion (*p* < 0.001). Microvascular inflammation (MVI) remained stable. As for the chronic lesions, all remained stable over time apart from the significant increase in tubular atrophy (*p* = 0.04).

**TABLE 5:** Comparison of histological lesions before and after conversion.

Variables	Before Switch, *n* = 48	After Switch, *n* = 48	*p*-value
Time in days (median, IQR)	28 (9–71)	378 (182–802)	—
Acute tissue injury			
Banff lesions score ≥1 in at least one compartment, N (%)	23 (47.9)	30 (62.5)	—
Acute tubular necrosis, N (%)	8 (16.7)	8 (16.7)	1
Glomerulitis (g), N (%)	4 (8.3)	5 (10.4)	1
Interstitial inflammation (*i*), N (%)	3 (6.2)	7 (14.6)	0.34
Tubulitis (*t*), N (%)	3 (6.2)	10 (20.8)	0.07
Peri-tubular capillaritis (cpt), N (%)	3 (6.2)	8 (16.7)	0.13
MVI (g + cpt ≥2), N (%)	0 (0)	1 (2.1)	1
Thrombotic microangiopathy, N (%)	11 (22.9)	0 (0)	<0.001
Chronic lesions			
Banff lesions score ≥1 in at least one compartment, N (%)	47 (97.9)	48 (100)	—
Transplant glomerulopathy (cg), N (%)	4 (8.3)	6 (12.5)	0.5
Interstitial fibrosis (ci), N (%)	43 (89.6)	48 (100)	0.06
Total inflammation (ti), N (%)	7 (14.6)	6 (12.5)	1
Tubular atrophy (ct), N (%)	40 (83.3)	47 (97.9)	0.04
Chronic vasculopathy (cv), N (%)	33 (68.8)	39 (81.2)	0.18
Arteriolar hyalinization (ah), N (%)	33 (68.8)	40 (83.3)	0.07
IFTA (ci + ct), median (IQR)	3 (2–3)	3 (2–3.25)	0.17

MVI, Micro-vascular inflammation.

## Discussion

We herein report a large monocentric cohort in a real-life situation where KT recipients were switched from CNI-based regimen to belatacept and followed for 3 years. In this cohort, belatacept safety was confirmed. At year 3, the recipient and death-censored kidney allograft survivals reached almost 90%. Estimated GFR improved significantly over the 36-month period after conversion, regardless of the switch early or late timing. To our knowledge, our study is the second-largest cohort studying CNIs-to-belatacept conversion as a rescue therapy with at least 3-years outcomes assessment, the longest follow-up currently available in this indication.

We observed a long-term benefit of CNIs-to-belatacept switch with significant improvement of kidney allograft function up to 3 years after the switch. Like other studies, eGFR significantly improved over the first 3 months, probably after suspending the hemodynamic effect of CNIs (8, 13, 14, 25), and remained stable up to year 5 after conversion in our study. Long-term benefits of belatacept in kidney allograft recipients treated with *de novo* belatacept and no CNIs are well known (9, 10). Recently, a similar benefit has been demonstrated at 24 months after the switch in kidney allograft recipients, regardless of time after transplant and cause of switch (9–11, 26, 27). However, our KTr were older, sourced their grafts mainly from ECD, and had lower eGFR (<35 ml/min/1.73 m^2^) (26). UPCR remained stable after conversion without worsening as already described in short-term follow-up studies (13). We also observed a long-term improvement of metabolic parameters such as the reduction in LDL cholesterol and HbA1C with stabilization of triglycerides concentration. Blood pressure remained stable after belatacept conversion. Other studies have already described such metabolic benefits (11, 28) and their short-term stability after CNI-to-belatacept conversion, our results confirmed the long-term stability (13). The clinical outcome of these metabolic changes needs to be further investigated in much longer-term studies.

In the whole cohort and in the late switch group, the leading cause of conversion was histological chronic vascular lesions associated with non-optimal kidney allograft function, whereas in the early switch group it was prolonged DGF. This real-life study design is different from that used in other studies which relied only on patients with stable eGFR (35–75 ml/min/1.73 m^2^) (16). Here we confirmed that belatacept is a useful immunosuppressive agent at any time after transplantation and for any cause, even in patients with poor prognostic clinical features.

Recipient and kidney allograft survivals were up to 90% at year 3 after conversion and belatacept safety remained acceptable. Early and late switch groups had similar survivals, suggesting that belatacept could increase kidney allograft survival at any time after transplantation as in KTr with severe vascular lesions (15). Survival results reported in other studies varied according to KT recipients’ characteristics (14, 16). In ours, age was the sole significant post-switch risk factor for death. eGFR level at switch was not a risk factor for neither death nor for graft loss, suggesting that conversion could be beneficial in all patients irrespective of their eGFR level. Age should be considered in the clinical decision and further research is warranted to investigate the effects of belatacept conversion in the elderly (i.e., > 70 years old).

OIs incidence in our cohort was comparable to previously published cohorts (29, 30). Alike for OIs leading causes: CMV disease and pneumocystis pneumonia (29,30). Accordingly, we suggest maintaining CMV and Pneumocystis pneumonia prophylaxis in early conversion, close monitoring of CMV viremia, and considering pneumocystis pneumonia prophylaxis in case of lymphopenia (lymphocytes count <1,000/mm^3^) (31). Infectious risk should always be considered upon deciding to switch. Similar to other studies, the incidence of malignancies, as well as the low occurrence of PTLD, confirm the low risk of malignancies after belatacept treatment (16).

This is the largest cohort that focuses on kidney allograft histological evolution after conversion from CNI to belatacept. Around 60% of the second biopsies were for-cause. We observed TMA disappearance with no development of ABMR. The usefulness of CNI-to-belatacept conversion in patients with TMA has been described in a few case reports (32–35). In our work, more than 10 TMA lesions vanished after switching to belatacept, suggesting that the latter alone might satisfy cost-effectiveness standards and be a safer strategy than if coupled with Eculizumab in recipients with TMA lesions (33). Post-switch biopsies showed no worsening in MVI (g + cpt ≥2) but precautions should be taken given the higher risk of allograft loss in these patients (36). Regarding chronic damages, we did not find significant variation over time except for tubular atrophy alone. Nevertheless, interstitial fibrosis and tubular atrophy (IFTA) remained stable, contrary to their tendency to worsen as described in a cohort of post-switch surveillance biopsies (37). Clinical outcomes such as eGFR might be a better predictor of graft outcomes as compared with IFTA (*p* = 0.031), which is consistent with eGFR improvement after belatacept conversion in our cohort (38). More data on CNI group comparison are still needed to assess kidney allograft histological modifications after conversion.

Considering immunological risks, switching our KTr to belatacept appeared to be safe as the prevalence of rejection was 10%. Similar results were observed in former studies even in sensitized patients (14, 16). Acute rejection appeared quickly after conversion and almost all episodes were TCMR. Short CNI association could be considered especially in early conversion to avoid acute TCMR rejection risk (39). Despite the acute rejection, allograft renal function improved significantly after 3 years. We also confirmed the low incidence of ABMR in recipients with preformed DSA treated with belatacept. DSA detected before the switch remained stable irrespective of other parameters and the incidence rate of *dn*DSA was null over 5 years after conversion. A similar 7-years incidence has been reported in BENEFIT and BENEFIT-EXT studies with higher MFI thresholds (i.e., > 2,000) (40, 41). The post-switch incidence of *dn*DSA was similar to the former study (27). DSA detection techniques and thresholds vary and can explain the differences in results (16, 17, 42). The low incidence of *dn*DSA with belatacept might be explained by the modulation of B-cell function, directly and at the level of B cell-Tfh interaction, incurring impairment of germinal center formation and improper antibody response in belatacept-treated KTr (43).

Despite some limitations including the monocentric, retrospective design and lack of control cohort, our study has several strengths: 1) 3-years post-switch follow-up in a real-life study design, 2) including recipients with impaired kidney allograft function who were potentially not eligible for randomized studies, 3) extensive data collection for each patient, from clinical characteristics to histological and DSA evolution. Data collection was exhaustive over a long follow-up interval.

In conclusion, we showed that in real-life conditions, conversion from CNIs to belatacept, as rescue therapy, is safe and beneficial in terms of long-term kidney allograft preserved function. Patient and kidney allograft survivals were excellent 36 months after conversion with a low incidence of SAE (acute rejection or infections). The immunological risk remained stable after conversion. CNI-to-belatacept switch should also be considered in CNI-treated recipients who develop TMA without ABMR, and could stabilize chronic histological lesions. Prospective studies are warranted to confirm those results.

## Data Availability

The raw data supporting the conclusion of this article will be made available by the authors, without undue reservation.
