# Supplementary material for: Three-Year Outcomes in Kidney Transplant Recipients Switched From Calcineurin Inhibitor-Based Regimens to Belatacept as a Rescue Therapy
Source: Transpl Int. 2022 Apr 13;35:10228. doi: 10.3389/ti.2022.10228 (PMC9043102; doi:10.3389/ti.2022.10228)
Supplement: Supplementary file 1 [file DataSheet1.docx]

**Table supp 1: Evolution after conversion. Major events Cause-Specific Hazard Ratio (CSHR), for death and allograft loss**

|  | **HR for deaths** | **p-value** | **HR for allograft loss** | **p-value** |
| --- | --- | --- | --- | --- |
| Early or late switch groups |  |  |  |  |
| Late switch | 1 |  | 1 |  |
| Early switch | 1.36 [0.51-3.63] | 0.54 | 0.88 [0.24-3.25] | 0.84 |
| Gender |  |  |  |  |
| Female | 1 |  | 1 |  |
| Male | 0.78 [0.30-2.02] | 0.61 | 0.51 [0.16-1.59] | 0.24 |
| Age |  |  |  |  |
| Age at conversion (per 1Y increase) | 1.05 [1.01-1.1] | **0.01** | 1.03 [0.99-1.08] | 0.12 |
| Renal function |  |  |  |  |
| eGFR (ml/mn/1.73m^2^) | 1.02 [0.99-1.04] | 0.18 | 0.98 [0.95-1.02] | 0.39 |
| eGFR < 30 ml/mn/1.73m^2^ | 0.81 [0.32-2.04] | 0.65 | 0.81 [0.26-2.51] | 0.71 |

**1Y:** 1 year ; **eGFR:** Estimated glomerular filtration rate (MDRD)

**Table supp 2: eGFR and UPCR evolution at different timepoints, with paired t-test analysis, with and without sensitivity analysis**

| **eGFR mL/min/1.73m²** | **At switch** | **3 months N=104** | **12 months N=86** | **24 months**  **N=76** | **36 months**  **N=62** | **48 months**  **N=42** | **60 months**  **N=34** |
| --- | --- | --- | --- | --- | --- | --- | --- |
| Whole cohort  mean (SD) | 31.5 (17.5) | 37.0 (17.5) | 36.7 (13.7) | 36.4 (14.5) | 36.7 (15.7) | 37.3 (16.3) | 40.1 (16.2) |
| Paired mean of the differences* |  | + 5.6 | + 5.4 | + 5.0 | + 5.4 | + 5.4 | + 5.5 |
| P value |  | **<0.01** | **<0.01** | **<0.01** | **<0.01** | **<0.01** | **0.01** |
| Sensitivity analysis** |  | N=105 | N=89 | N=80 | N=67 | N=48 | N=41 |
| Whole cohort  mean (SD) | 31.5 (17.5) | 36. 7 (17.7) | 35.6 (14.6) | 34.9 (15.7) | 34.4 (17.1) | 33.4 (18.5) | 34.3 (19.7) |
| Paired mean of the differences* |  | + 5.6 | + 4.7 | + 4.2 | + 3.9 | + 2.5 | + 1.2 |
| P value |  | **<0.01** | **<0.01** | **0.01** | 0.05 | 0.25 | 0.62 |
| Sensitivity analysis*** |  | N=109 | N=98 | N=90 | N=81 | N=67 | N=61 |
| Whole cohort  mean (SD) | 31.8 (17.5) | 35.6 (18.3) | 32.9 (16.3) | 31.7 (17.3) | 29.5 (19.0) | 25.6 (20.0) | 25.0 (20.9) |
| Paired mean of the differences* |  | + 3.7 | + 1.0 | + 0.1 | -3.2 | -8.1 | -10.1 |
| P value |  | **0.02** | 0.61 | 0.97 | 0.24 | **0.01** | **<0.01** |

* Paired mean of the difference with reference mean at time of switch

** Analysis with imputation of missing values of allograft loss: 6 mL/min/1.73m²

*** Analysis with imputation of missing data of allograft loss and death: 6 mL/min/1.73m²

**Table supp 2 (continued)**

| **Urine protein/creatinine**  **Ratio (UPCR)** | **At switch** | **3 months N=102** | **12 months N=86** | **24 months**  **N=76** | **36 months**  **N=62** | **48 months**  **N=43** | **60 months**  **N=34** |
| --- | --- | --- | --- | --- | --- | --- | --- |
| Whole cohort  UPCR > 100 mg/mmol  N (%) | 31 (27) | 21 (20.6) | 15 (17.4) | 16 (13.9) | 12 (10.4) | 7 (6.1) | 4 (11.8) |
| P-value |  | 0.75 | 0.15 | 0.45 | 0.21 | 0.11 | 0.22 |

**Table supp 3: Metabolic parameters evolution 36-months after conversion**

| **Variables** | **At time of switch** | **At 36 months** | **p-value** |
| --- | --- | --- | --- |
| Anti-hypertensive drugs, median (IQR) | 2 (1-2) | 2 (1-2) | 0.87 |
| HbA1C (%), mean ± SD | 5.9 (0.5) | 5.7 (0.4) | **<0.01** |
| LDL-c (g/L), mean ± SD | 2.1 (0.5) | 1.9 (0.5) | **<0.01** |
| HDL-c (g/L), mean ± SD | 1 (0.3) | 0.9 (0.3) | **<0.01** |
| Triglycerides (g/L), median (IQR) | 1.4 (1.2-2.2) | 1.8 (1.2-2.3) | 0.39 |

**Table supp 4: Comparison of patients characteristics according to eGFR trajectory clusters**

| **Variables** | **Cluster A**  **N=64** | **Cluster B**  **N=50** |  |
| --- | --- | --- | --- |
| ***Recipient caracteristics*** |  |  |  |
| Age, mean ± SD | 56.4 (15.6) | 56.0 (14.6) | 0.88 |
| Male gender, N (%) | 38 (59.4) | 37 (74.0) | 0.10 |
| Hemodialysis, N (%) | 63 (98.4) | 42 (84.0) | **0.01** |
| Previous kidney transplantation, N (%) | 13 (20.3) | 2 (4.0) | **0.01** |
| Initial nephropathy |  |  | 0.80 |
| Glomerulopathy, N (%) | 12 (18.8) | 12 (24.0) |  |
| Diabetes mellitus, N (%) | 9 (14.1) | 9 (18.0) |  |
| Hypertension, N (%) | 8 (12.5) | 3 (6.0) |  |
| Genetic, N (%) | 5 (7.8) | 5 (10.0) |  |
| Autoimmune disease, N (%) | 1 (1.6) | 2 (4.0) |  |
| Other, N (%) | 14 (21.9) | 8 (16.0) |  |
| Undetermined, N (%) | 15 (23.4) | 12 (24.0) |  |
| ***Donor caracteristics*** |  |  |  |
| Age, mean ± SD | 62.6 (14.2) | 60.0 (16.0) | 0.34 |
| Extended criteria donor, N (%) | 41 (64.1) | 27 (54.0) | 0.27 |
| ***Switch*** |  |  |  |
| Early, N (%) | 21 (32.8) | 17 (34.0) | 0.89 |
| eGFR mL/min/1.73m², mean ± SD | 26.4 (14.6) | 36.4 (15.8) | **<0.01** |
| eGFR <30 mL/min/1.73m², N (%) | 49 (76.6) | 17 (34.0) | **<0.01** |
| ***Kidney biopsy (Banff lesions score)*** |  |  |  |
| Acute tissue injury |  |  |  |
| Acute tubular necrosis, N (%) | 12 (20.3) | 8 (18.2) | 0.78 |
| Glomerulitis + Peri-tubular capillaritis ≥ 2, N (%) | 1 (1.7) | 2 (4.5) | 0.60 |
| Acute thrombotic microangiopathy, N (%) | 10 (16.9) | 10 (21.3) | 0.30 |
| Chronic lesions |  |  |  |
| Transplant glomerulopathy, N (%) | 5 (8.5) | 3 (7.0) | 1 |
| Interstitial fibrosis + Tubular atrophy, N (%) | 47 (82.5) | 34 (79.1) | 0.66 |
| Chronic vasculopathy ≥ 2, N (%) | 21 (36.2) | 18 (41.9) | 0.33 |
| Arteriolar hyalinization + Chronic vasculopathy ≥ 2, N (%) | 46 (80.7) | 31 (72.1) | 0.31 |
